# The Design of an Energy Harvesting Wireless Sensor Node for Tracking Pink Iguanas

**DOI:** 10.3390/s19050985

**Published:** 2019-02-26

**Authors:** Pierpaolo Loreti, Alexandro Catini, Massimiliano De Luca, Lorenzo Bracciale, Gabriele Gentile, Corrado Di Natale

**Affiliations:** 1 Deptartment of Electronic Engineering, University of Rome Tor Vergata, Via del Politecnico 1, 00133 Rome, Italy; catini@ing.uniroma2.it (A.C.); lorenzo.bracciale@uniroma2.it (L.B.); dinatale@eln.uniroma2.it (C.D.N.); 2 CNR—Institute of Marine Engineering, Via di Vallerano 139, 00128 Rome, Italy; massimiliano.deluca@idasc.cnr.it; 3 Department of Biology, University of Rome Tor Vergata, Via della Ricerca Scientifica 1, 00173 Rome, Italy; gabriele.gentile@uniroma2.it

**Keywords:** energy harvesting, IoT, animal monitoring, GPS tracking, wireless device

## Abstract

The design of wireless sensor nodes for animal tracking is a multidisciplinary activity that presents several research challenges both from a technical and a biological point of view. A monitoring device has to be designed accounting for all system requirements including the specific characteristics of animals and environment. In this work we present some aspects of the design of a wireless sensor node to track and monitor the pink iguana of the Galápagos: a recently discovered species living in remote locations at the Galápagos Islands. The few individuals of this species live in a relatively small area that lacks of any available communication infrastructure. We present and discuss the energy harvesting architecture and the related energy management logic. We also discuss the impact of packaging on the sensor performance and the consequences of the limited available energy on the GPS tracking.

## 1. Introduction

Tracking devices are fundamental tools to study the behavior of wild animals in relation to their natural environment. Retrieved information can guide the design of effective strategies and actions aimed at protecting endangered species and optimizing ecosystem management. Thus, in the last years, such rationale has prompted the design and the development of a plethora of non-commercial and commercial monitoring devices, each characterized by a trade off between biological requirements and technical limitations [1]. The selection of suited technologies requires a tight collaboration between engineers and wildlife conservationists: for instance, the choice of a specific network technology may have strong implications on the size of the device, and power consumption which may have a reflection on the weight and size of the battery loaded on the animal. This ultimately affects the duration of monitoring. In the last years, technological advances enabled the development of a new generation of tracking devices: smaller, more energetically efficient, more ecologically sound (“green”), and thus best suited to monitor animals in the wild.

In this work we present some design aspects of a device aimed at characterizing the long-term behaviour of Galápagos pink land iguanas (*Conolophus marthae*, hereafter pink iguanas): a recently discovered species of iguana [2] that is assessed as *Critically Endangered*. Prior attempts to investigate the behavior of this animal were ineffective due to a combination of the difficulty of the terrain and hard logistics that limits the duration of field trips, lack of suitable tracking devices, and absence of a network infrastructure in the interested area. The harsh environmental conditions require a environmental-friendly, water-resistant, low-power, wireless system.

For these reasons, we developed a wireless sensor node that integrates several elementary sensors, a GPS receiver and storage capabilities. The device is powered by an energy harvesting circuit composed by a small-size solar cell combined with a super-capacitor in order to power the energy-consuming GPS receiver. The system is complemented by an high power long range communication transmitter. The combination of energy harvesting and long-range communication should theoretically enable a perpetual monitoring [3]. The optimization of the energy requirement and harvesting can balance the need to limit the size of the device (to avoid interference with animal habits) and the opportunity to collect, alongside GPS data, environmental parameters such as temperature, humidity, UVB radiation, etc.

We also pursued a green engineering approach by using processes and products to minimize potential hazards and toxicity, including, for example, super-capacitors that have an impact on the environment lower than Li-Ion batteries [4]. This objective represents an important advance in this field and it is of paramount importance in this application where devices are going to be deployed in natural sanctuaries and remote settings where the probability of not recollection is high.

The main goal of the paper is to demonstrate how current sensor technology can be effectively used to solve a real zoology problem such as tracking the pink iguana in a harsh environment. The main contributions of this work are:

- The description of the tracking problem in an harsh environment with a specific reference to the Galápagos Pink Iguana;
- The energy harvesting and power architecture solution, that enables long distance communication by powering both an high current demanding RF booster and the energy demanding GPS module;
- The description of the device packaging and of the real deployment on the field;
- An analysis of power consumption, sensors calibration and GPS fixing performance.

The paper is organized as follow: in Section 2 we briefly introduce the biological problem and the pink iguana habitat; Section 3 describes the selected hardware components, the energy harvesting and power architectures adopted and the enclosure and attachment solutions; Section 4 and Section 5 report the sensors’ calibration and the energy consumption tests respectively; in Section 6 we report the related work also comparing the device with those commercially available.

## 2. The Pink Iguana Tracking Problem

**Figure 1:**
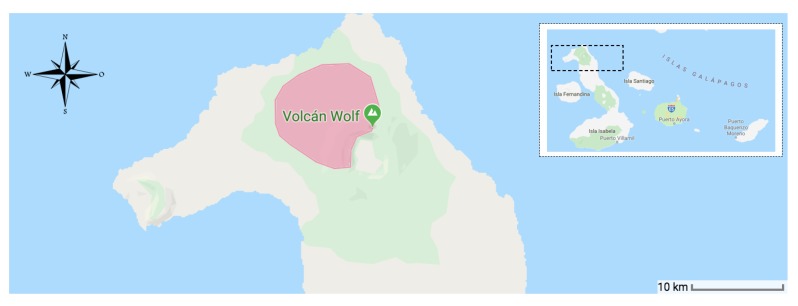
Map of the monitoring field.

The existence of the new iguana species was reported to Science in 2009 [5]. Since then, pink iguana rapidly became a flagship species, internationally recognized in Science, Arts, and Society [6]. The species, which has substantial evolutionary legacy [7]), is endemic to Wolf Volcano on the Isabela island (Galápagos, Ecuador), at altitudes ranging from 600–1700 m asl. It lives in syntopy with a population of *C. subcristatus*, another endemic species of iguana, and the two species do not hybridize. There are several issues that threaten the existence of pink iguanas. These include the small population size (census N=192; Ne=90.7), the limited area of distribution (less than 25 ${\mathrm{km}}^{2}$) depicted in Figure 1, the possible competition with syntopic species, and finally the presence of introduced predators (feral cats and black rats). In 2012, this species was included in the International Union for Conservation of Nature (IUCN) Red List, under the category Critically Endangered [8].

The difficult access to the site limits the duration of field studies. Thus, current knowledge about the reproductive biology of both the syntopic species in Wolf Volcano is scarce. Ultrasound and hormonal surveys, performed when the species are sexually active, indicate that only a few *C. marthae* females reproduce, with *C. marthae* showing a smaller clutch size than *C. subcristatus* [9]. Since 2005, no hatchlings, only one juvenile, and a few subadults was observed. This strongly suggests that population recruitment may be non-effective. For these reasons, the Galápagos National Park Directorate, the legal authority that governs the biodiversity of Galápagos islands, is considering a head-start program to increase the number of pink iguanas.

The habitat of pink iguanas is found on the top and along the slopes of Wolf Volcano, a harsh environment characterized by small craters, canyons and thick vegetation. The area includes tropical dry shrub land at the top of the volcano and tropical dry forest along the slopes. Temperature at the highest altitudes may range from approximately 10 C (night) to 35 C (day). Vegetation may be impacted by droughts. It must be noted that the harsh terrain and difficult logistics of the area do not allow the use of long-term traditional radio-tracking techniques. Attempts to use GPS devices on land iguanas were carried out in the early 2000 s, and proved unsuccessful mainly due to the large size of the smallest GPS equipment available and difficult logistics. However, the advance of technology and miniaturization of components allows us to devise small devices that better fit the requirements of a pink iguana monitoring scenario.

## 3. Device Design

The architecture of the sensor node includes: electronic design, energy harvesting and power management, and packaging. In the following sections we illustrate each of these aspects.

### 3.1. Electronics Design

**Figure 2:**
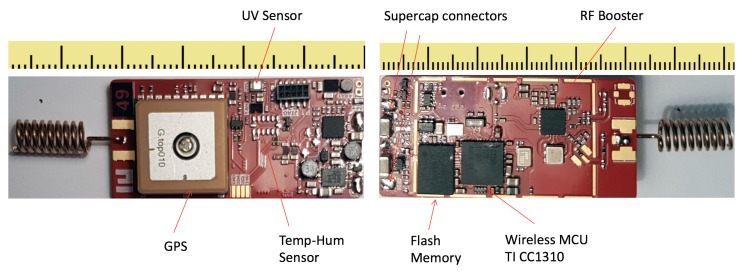
Sensor board components placement.

Figure 2 shows board layout and components placement. The operations of the sensor node are the following: (i) acquisition GPS position and (ii) retrieval of sensors values, (iii) storing all information and (iv) data transmission through the gateway. The fulfillment of these functions relies on the following electronic components:

1. CC1310 wireless controller [10], an ultra-low-power device from Texas Instrument with a low active RF and MCU current consumption and flexible low-power modes. It provides excellent battery life and allows long-range operation. It integrates a powerful 48-MHz Arm Cortex-M3 micro controller, a dedicated Radio Controller and a ultra low power Cortex M0 sensor controller.
2. Sensor set including an accelerometer (ADXL345 [11]), a digital humidity sensor with integrated temperature sensor (HDC1008 [12]) and a UV light sensor (VEML6070 [13]);
3. Global TOP PA6C GPS receiver based on MediaTek MT3329 chipset and an integrated ceramic antenna. It is characterized by a larger receiver sensitivity (−165 dBm thanks to the TCXO Design) resulting in an high position accuracy.
4. NOR flash memory storage with a capacity of 1 GB (MT25QL256ABA8E12).
5. Skyworks SE2435L [14]. This is a high performance RF front-end module operating in the 860–930 MHz frequency band that includes a power amplifier for transmission and a Low Noise Amplifier for reception.

**Figure 3:**
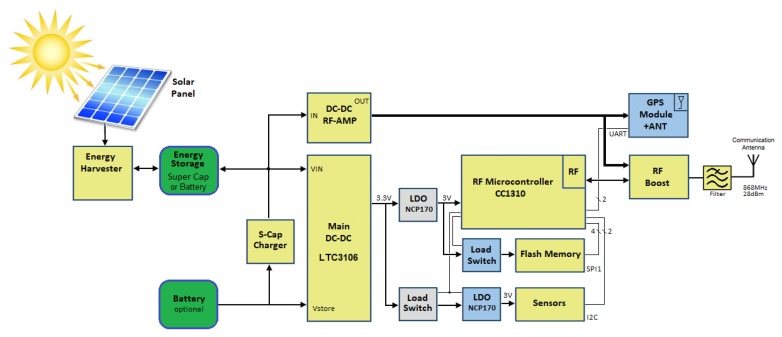
Node Architecture.

The overall node architecture is shown in Figure 3. All the functions are coordinated by the CC1310 controller that implements the node logic. The GPS receiver provides the position of the node; it communicates with the controller through a UART interface. The sensor set delivers data to the controller by a I2C interface. Finally, a SPI interface accesses to the flash storage memory. The CC1310 integrates a wireless modem that directly generates and receives RF signals. To improve the node communication range, all the signals go through the Skyworks RF front-end module and a band-pass filter.

The device integrates solar panels and super-capacitors (for energy storage) as key enabler to an almost perpetual operations of the wireless sensor node. To this regard, the DC-DC conversion can operate from the range of nanowatts, in deep sleep mode, up to Watts in the long range transmission mode (the SE2435L absorbs 250 mA with 23 dB of gain). Moreover, the CC1310 implements the Wake-on Radio technology. This feature embeds a low power receiving mode that can enable the discovery functionality of the devices while in sleep mode [15].

### 3.2. Energy Harvesting and Power Architecture

As shown in Figure 3 the electric charges generated by the solar cell micro panels are accumulated in a super-capacitor. The charge of the super-capacitor is provided by the series of a harvester power management integrated circuit (BQ25570) and a super-capacitor charger (LTC3225). The BQ25570 can extract very small amount of power (down to microwatts) generated by the solar panels while the LTC3225 is designed to charge, at a selectable fixed output voltage, two super-capacitors connected in series. Automatic cell balancing prevents overvoltage damage.

A software-controlled dual path DC-DC converter (LTC3106) manages the flow of charges from the super-capacitor to and from the battery. The super-capacitor is the primary power source. The LTC3106 can also charge the backup battery whenever an energy surplus is available. In absence of a load, the LTC3106 generates an output voltage up to 5 V from the input sources with a very low power stand-by consumption. When the solar power is unavailable, the LTC3106 switches to an alternative power sources such as the battery. Despite the device is ready for battery integration, for the specific Iguana tracking problem in the Galapagos Islands, the battery can not be used for environmental reasons.

The output of the LTC3106 is affected by a ripple [16] that results in a power noise that propagates through the microcontroller and the RF oscillator. Eventually the ripple results in a noisy communication. This effect is counteracted by the low drop-out (LDO) voltage regulator (NCP170) placed at the output of the LTC3106.

To better control energy consumption, the board is divided in four independently powered sections corresponding to the GPS, the RF front-end, the sensor set and the flash memory respectively. The controller can selectively power each section according to the application requirements and the available energy. Additionally, the power control unit makes use of a high drain line to supply power to the RF booster. Specifically, the high drain line can provide up to 1 A of current to the Skyworks SE2435L power amplifier in order to achieve the largest RF gain. It is worth to note that the maximum output power can be dynamically controlled by the CC1310 microcontroller through a DAC that can change the supply current of the SE2435L module. The rationale of this line split is the presence of the booster that in turn calls for an high current drain that allows long range transmissions.

### 3.3. Device Packaging and Animal Fixture

**Figure 4:**
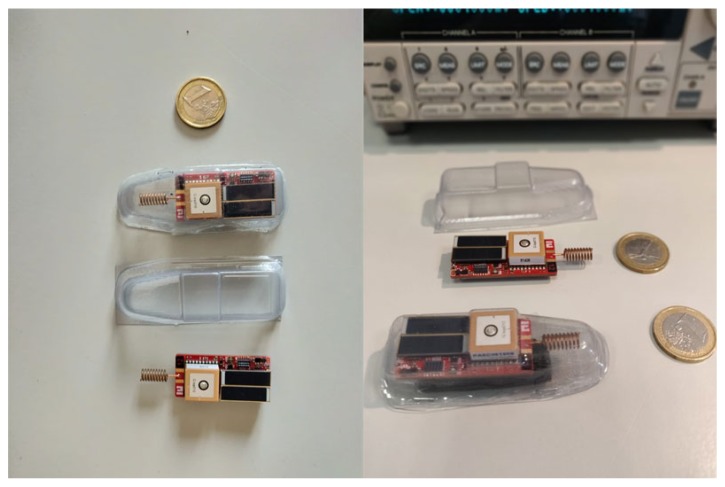
WSN inside and outside the packaging.

To protect the device against environmental impacts (scratches, bumps, breakages, and pouring water), a PVC (PolyVinyl Chloride) package, shown in Figure 4 has been designed. Besides offering efficient protection to the hardware and to provide an easy and safe attachment on animal skin, the packaging should also have a null impact on sensors and on the energy harvester. For the accelerometer, a tight connection of the board to the package and then to the animal skin is required in order to avoid any interference due to the vibration of the board inside the packaging. Regarding to UV sensor and solar cells the obvious requirement is the transparency in the relevant wavelength ranges.

The board packaging was molded in two separate parts from a 3 mm thickness transparent PVC. The lower base of the container is concave to facilitate the adhesion to the back of the animal, while the lid is shaped to adapt to the board profile. The two parts are tightly sealed through a thermoforming process. The packaged system has been mechanically tested to simulate stress due to the rubs occurring when iguanas digs their burrows and/or move inside them. It’s worth to mention that the material used for the case is transparent furniture grade PVC treated to be UV and high impact resistant. This means that the box does not get brittle or brownish under prolonged sun exposure.

**Figure 5:**
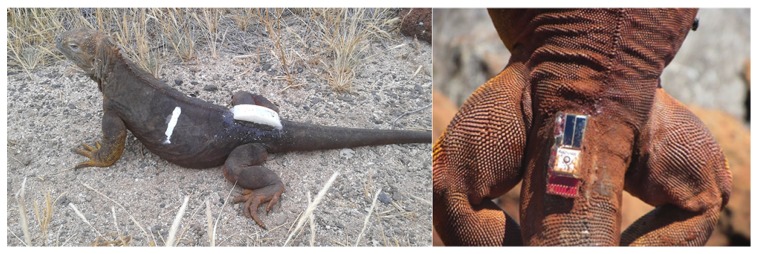
On the left, animal with the device glued. On the right, the attachment of the WSN on the iguana back.

Special attention has been devoted to the attachment of the node to the animal. Whereas the device could be surgically implanted, we preferred not to consider microsurgery because it may cause animal stress and requires a proper environment to be performed. Other methods based on fastened jackets were not considered as they also proved unsafe to iguanas in long term studies [17]. We instead performed several attachment tests by experimenting gluing a box (Figure 5) and gluing the board protected by a synthetic resin. In a pilot test, we showed that epoxy glue ensures that the device remains attached for at least eleven months. We are aware that shedding skin could in theory cause the detachment of the device. However we preferred to accept such a potential risk rather than increase the risk to harm iguanas by using more invasive methods.

## 4. Sensors Calibration

In this section we present the various issues we tackled calibrating the different sensors available on the board. Accelerometer and GPS have been used on the basis of the characteristics provided in the respective datasheet. Thus, no specific calibration has been performed for these devices. Different was the case of the UV sensor, in this case, a possible UV attenuation by the PVC case could occur and then the actual correspondence between the signal provided by the sensors and the intensity of the radiation in the UVB band has been measured. In previous reptile studies [6] the UV-B intensity was measured with a Digital Ultraviolet Radiometer (Model 6.2201UVB) made by Solartech Inc. This is a portable instrument based on a silicon carbide photodiode which is rather insensitive to the UVA spectral band with a peak of responsivity in the range 240–320 nm [18]. This device was shown to be sufficiently accurate for the scope and it has been used as reference to test the embedded sensor.

**Figure 6:**
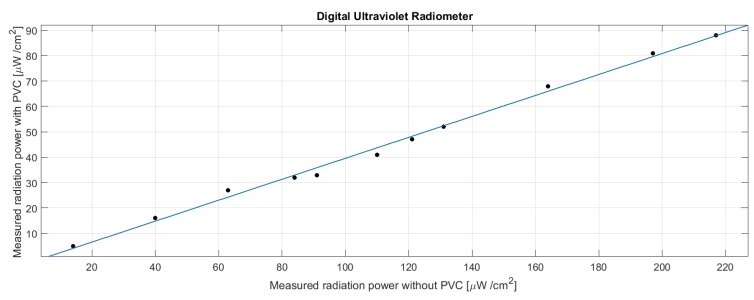
Relationship between the intensity of UVB detected by the digital ultraviolet radiometer outside and inside the PVC case. Each point corresponds to a different time of the day. Linear fit is also plotted.

The digital ultraviolet radiometer has been used to measure the transmission of light in the UVB band of PVC during exposure to sunlight. Measurements were performed in sunny days in Rome at a latitude of approximately 41° North. It is worth to consider that Galapagos islands are close to the equator with a latitude of about −0.7°. Figure 6 shows the power intensity (W/m${}^{2}$) measured exposing the ultraviolet radiometer to the direct sunlight versus the power intensity measured behind the PVC case. Different intensities correspond to different hours of the day.

The attenuation is of the order of 50%; however, the transmission is rather constant in the range from 20 to 200 W/m${}^{2}$ of incident radiation. The linear regression of the collected data provides the estimation of the intensity in the UVB range from a measure of the light intensity from the inside of the case. The relationship between measured and estimated intensities is $I=A\cdot I_{meas}+B$ where A=2.42±0.02 and B=2.51±1.25. The ratio of the intensity of the solar radiation in the UVA (λ = 320–400 nm) and UVB (λ = 280–320 nm) ranges has been evaluated to be about 20 for most of the part of the day, and in particular when the elevation of the sun is larger than 60° [19]. At low elevations, due to the atmospheric refraction, the ratio quickly increases exponentially. Considering that the absorption of UVA is negligible in PVC and the ratio of UVB/UVA of the solar light is constant for a long part of the day, instead of measuring directly the UVB intensity attenuated by PVC, we decided to measure the more intense UVA radiation and to estimate the UVB intensity. For the scope a UVA detector (VEML6070 Vishay Semiconductors) has been used. This is an integrated UV sensor that besides the photodiode include, in a single chip, also the amplifier and the digital interface. The VEML 6070 is temperature compensated and shows a linear responsivity to the UV light with a saturation at 328 mW/cm${}^{2}$. The peak of responsivity lies in the UVA range.

**Figure 7:**
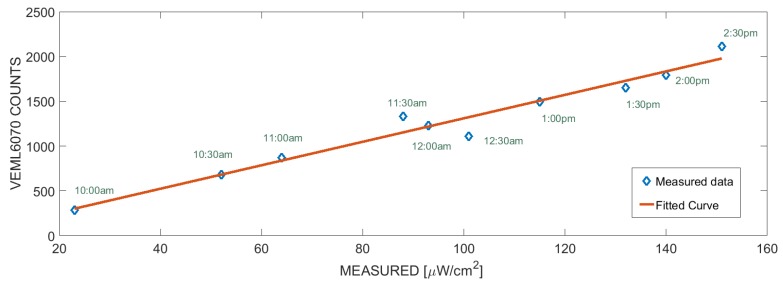
Calibration curve of VEML6070 sensors at different daytime, the measured intensity of UVB has been obtained by an ultraviolet radiometer. Linear fit is also shown.

**Figure 8:**
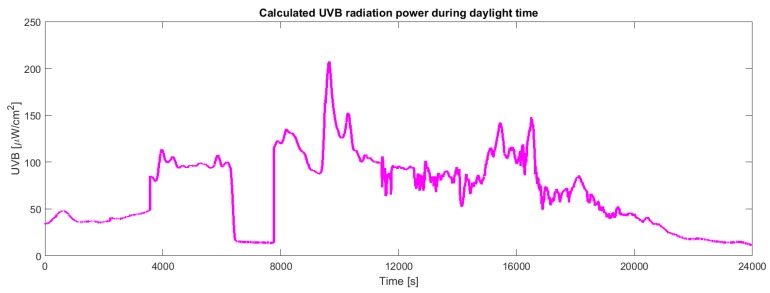
Behaviour of the UVB estimated from the VEML6070 sensor. Data have been continuously collected during a day.

The VEML6070 UV light sensor has been calibrated with respect to the UVB intensity measured by the ultraviolet radiometer. For the scope, the sensor and the ultraviolet radiometer have been exposed to the sunlight. Results show a good reproducibility at different intensities of sunlight and at different weather conditions. Figure 7 shows the calibration data fitted by a straight line. The linear fit achieved $R^{2}=0.96$. The UVB intensity has been estimated from the calibration curve. Figure 8 shows the behaviour of the intensity of UVB radiation in a partly cloudy day. Samples have been collected every 2 s during a typical partly cloudy day starting from 10:00 to 16:40.

**Figure 9:**
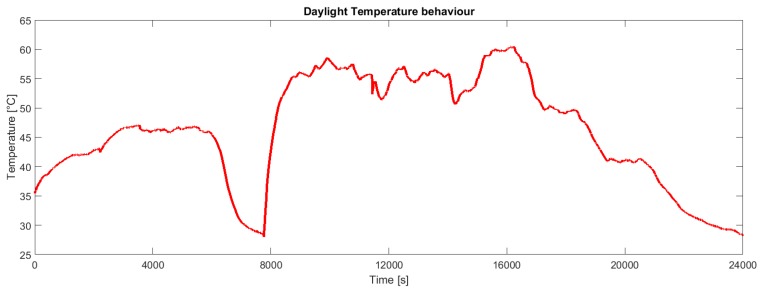
Continuous behavior of the board temperature measured.

**Figure 10:**
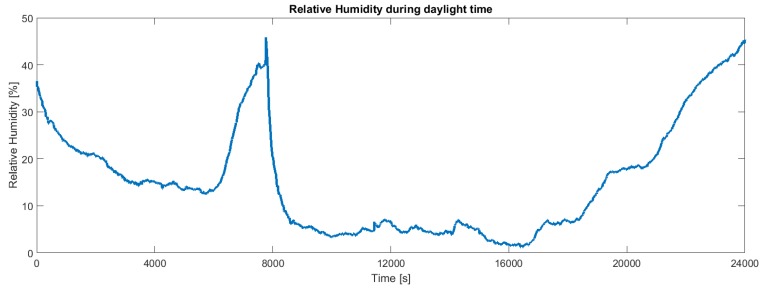
Behavior of the relative humidity corresponding to the temperature in Figure 9.

The calibration of the HDC1008 temperature and humidity sensor has been performed with respect to the measures provided by a Sensirion SHT21 digital temperature and humidity sensor. Note that the humidity sensor has been calibrated outside the package. Clearly, when the device is encapsulated, the sensor measures the humidity inside the capsule. As shown in Figure 9, even in a temperate climate, a continuous exposure to sunlight brings the temperature of the board above 60 °C. The relative humidity is obviously correlated with the temperature (Figure 10). Samples have been collected every 2 s on a partly cloudy day from 10:00 to 16:40.

**Figure 11:**
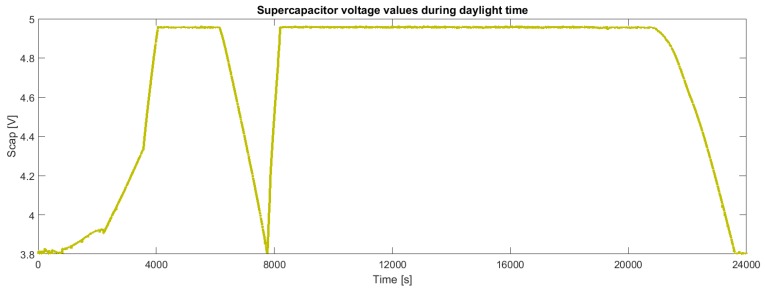
Behavior of the voltage of the supercapacitor during continuous operation.

Figure 11 shows the energy stored in the supercapacitors during a typical daytime. When the voltage falls down the board is draining energy because the power provided from solar panels is no more sufficient to perform all operations (for instance in case of cloudy weather or night period). For the same reason we can also observe from the other figures in the same period a UVB radiation and temperature decrease (Figure 8 and Figure 9) and an RH increase (Figure 10).

## 5. Energy Consumption Tests

In this section we present the experimental results about the energy consumption of the board with reference to its specific operating modes.

**Figure 12:**
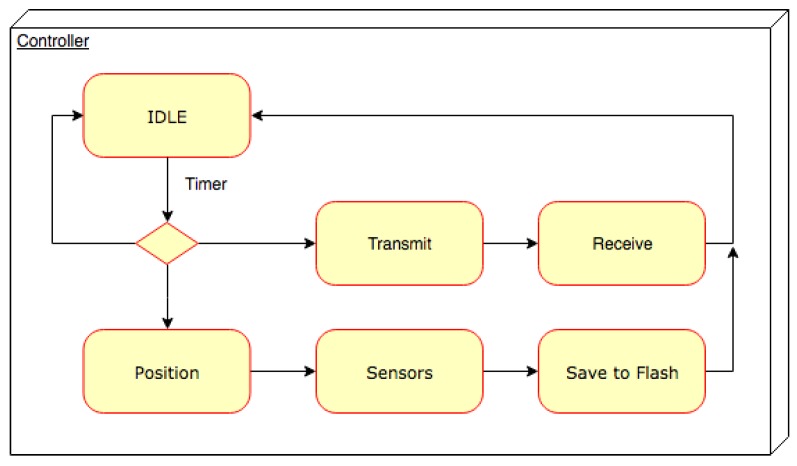
Simplified Finite State Machine of the controller.

Figure 12 shows the simplified finite state machine implemented in the CC1310 controller. In the IDLE state all power sections are switched off and the only power absorption is due to the LTC3106 and NCP170 LDO. The current absorbed by the main chip is 100 nA. In the position state, the GPS is active (absorbing 89.1 mW) during the attempts to acquire the position. Then, the sensors are activated and the data are collected and saved in the flash memory. Periodically, the system checks the flash memory to verify if there are still data to be transmitted. In this case, it attempts to transmit the data and waits, in the receiving state, for an acknowledgement packet. Table 1 reports the board average power consumption in the various states. The acquisition of the position by the GPS is the most power-consuming activity. The GPS unit needs a large amount of current to power the transceiver. Such high power consumption is caused by the fact that the chip has to be active for a long time, of the order of seconds, needed to increase accuracy in retrieving the position.

**Figure 13:**
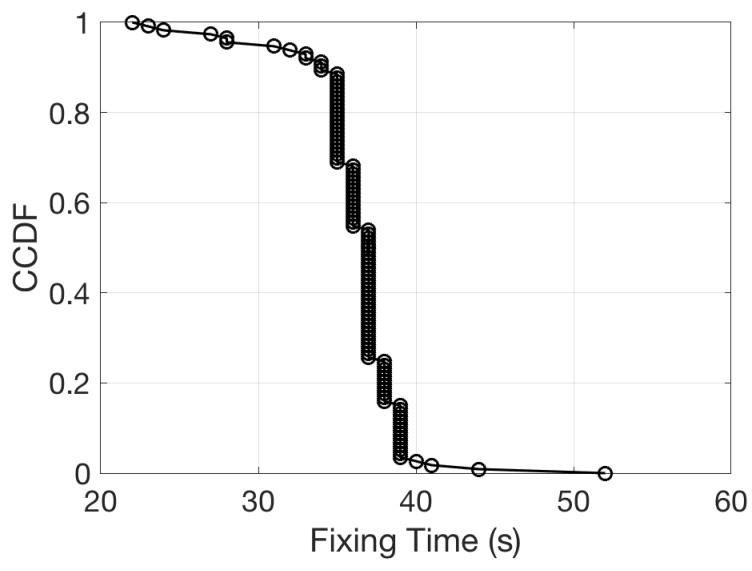
Complementary cumulative distribution function of the time to obtain the first fix from the GPS unit.

The time to retrieve the GPS position is then a crucial parameter for energy management. For this reason it has been carefully evaluated in a repeated procedure of position acquisition and reset. Measurements were collected placing the device in a open sky condition. The response time of the device has been defined as the time necessary to receive the first valid NMEA message of the type GPGGA from the GTOP PA6C GPS receiver (GPS fix). Figure 13 shows the complementary cumulative distribution function of the GPS response time. The distribution shows that about 40 s is the typical time necessary to get the first data, however, there is also a non negligible tail of the distribution that push this time up to 52 s.

**Figure 14:**
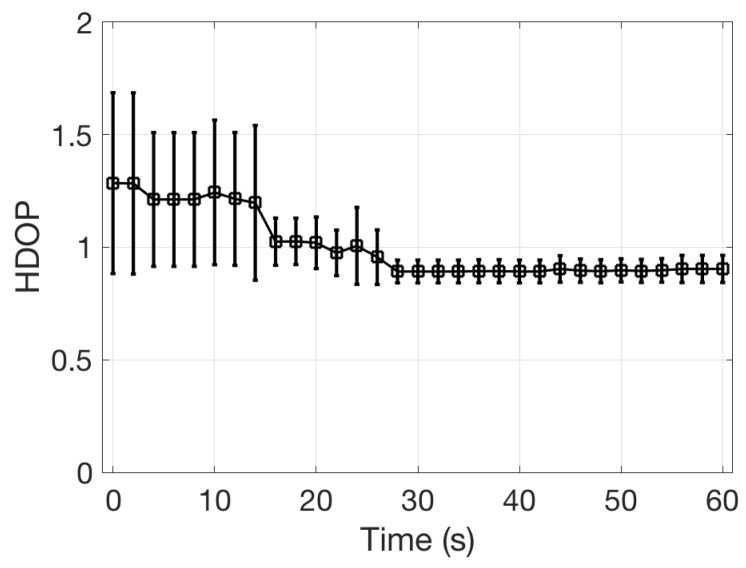
Distribution of the Horizontal Dilution of Precision (HDOP) as a function of the time from the first fix.

After the first fix, the accuracy of the position increases. The accuracy is measured by the HDOP parameter (Horizontal Dilution of Precision) [20] which is embedded in the NMEA message. In Figure 14 the value of the HDOP found in the tests is plotted as a function of the time from the first fix. A value of HDOP less than 1 is associated to the highest possible confidence level of the position estimation. The average of the HDOP after the first fix is only slightly larger than 1, however the wide dispersion in the parameter makes the first fix scarcely reliable. After 30 s from the first fix the mean value of the HDOP converges to 0.8 with a negligible dispersion.

The energy requirement of the GPS unit affects the dimension of the super-capacitor. The relationship between the capacitance, the delivered power and the voltage in off and on states are related by the following equation:

(1) $$C=\frac{tVI}{\eta (V_{\mathrm{ON}}^{2}-V_{\mathrm{OFF}}^{2})}$$

where *t* is the time when the GPS unit is supplied with a voltage *V* and a current *I*; η is the conversion efficiency from the current delivered by the super-capacitor to the current delivered to the GPS and the micro-controller, $V_{\mathrm{ON}}$ is the super-capacitor voltage at which the GPS is turned on, and $V_{\mathrm{OFF}}$ is the voltage at which the GPS in turned off.

In our case, V= 3 V, I=36 mA, η=0.95, $V_{\mathrm{ON}}=4.6$ V and $V_{\mathrm{OFF}}=3$ V. In clear sky conditions, at least 90 s are necessary for an accurate GPS position. To ensure the necessary power, the capacitance shoud at least 1.2F. This requirements are fulfilled byh the CAPX HS230 (1.20 F, 5.5 V) whose size is 39 mm × 17 mm × 3.9 mm.

## 6. Related Work

In this section we report some research works and projects that can be compared with the presented project and the developed device.

### 6.1. Animal Monitoring Devices and Projects

Several research projects tackled the design of wireless sensor network nodes for specific animal monitoring. Sometimes prototypes have also been developed and tested in the field. Among them, perhaps the most famous is project “Zebranet” [21], that in the early 2004 developed a collar for monitoring a herd of zebras. The wireless node uses a MSP430 microcontroller with an external 900 MHz wireless transponder Maxstream 9XStream that exhibits a coverage range of about 5 miles. The device is powered with a AA lithium battery, rechargeable with a solar panel. The resulting node is about 9 cm × 6 cm × 3 cm without antennas and solar panel. An extension of the Zebranet project is the wildCENSE project [22] that designs a wireless node as a cows collar. The wildCENSE node is based on an ATMega128 microcontroller and has an integrated Zigbee (IEEE 802.15.4) modem in 2.4 GHz ISM band that presents a power consumption higher than the 900 MHz transponder, but allows a higher data rate with a smaller antenna. Authors reported that the resulting device has a reduced board size of 6 cm × 5 cm, but the external battery is not accounted in this calculation. An interesting project was based on a multi-hop WSN designed for cattle [23]. The system is composed of a collar with an integrated a GPS receiver, an ATMega 128 controller and a Nordic NRF905 radio operating in the 915 MHz band. The system is powered by batteries recharged by solar cells. Similarly to the device presented in this work, that system uses UHF frequency band and data compression was implemented to reduce the energy consumption of the wireless communication. This software solution was necessary because the Wake-on Radio functionality was not available in the modem.

In [24] the LynxNet system, a new collar for lynxes is presented. It is based on a CC430 controller and a CC2420 433MHz radio modem. The prototype has been tested in an environment rich of vegetation and the radio showed a very short coverage range (about 250 m) with a packet loss ranging between 10% and 80%. A notably most recent contribution comes from the Mataki project [25] in which researchers developed an open hardware/open-source device based on a MSP430 microcontroller and a CC1101 modem operating in the 868MHz band. The resulting device is very small (43 × 21 × 7 mm, without the battery) but it does not integrate the harvesting component.

Another recent prototype of monitoring device for flying foxes is presented in [26]. This device is based on a CC430 wireless controller powered by a lithium battery charged by a solar panel. The communication range in this application is up to 200 m since the available transmission power is limited. However, the battery allows a longer GPS operation time and consequently an efficient estimation of the position. Many other works exist in literature, focusing on on specifics aspects of wireless node design. In [27], authors studied a device based on the IEEE 802.15.4 in the 900 MHz band for animal monitoring. The device does not integrate any GPS. It only includes sensors to monitor animal status, sending an alarm, via a wireless message, in case of alteration of some parameter. Authors produced a prototype using a Libelium Waspmote equipped with a Xbee modem. The main contribution of the paper is the decision algorithm to send the alarm. A similar work is presented in [28] where authors propose a system to rapidly detect animal’s illness using a IEEE 802.15.4 network in the 2.4 GHz bandwidth. They also studied the performance of the Zigbee technology in the case of a directional antenna that could communicate with a large number of devices. A different study is reported in [29]. They define a monitoring scenario for tigers and design a system based on a multihop IEEE 802.15.4 operating in the 2.4 GHz band. They simulate up to 2000 nodes in a 7 km × 25 km area and a UAV device that collects the information using the wireless channel.

We remark that all the analyzed solutions are based on the presence of relatively high capacity battery and do not use ultra long range communication technology, that requires the management of an high drain current by the RF amplifier. Conversely the presented solution relies on only one supercap of 1.2 F, powering all the components and in particular the GPS and an up to 28 db RF booster.

### 6.2. Energy Management and Energy Harvesting

The energy management architecture is a key component in the design of a wireless node that should work for a long time.

When the node is battery-powered the only solution is to use techniques aimed at limiting power consumption. In this case, a simple but effective technique is to limit the working intervals (duty cycle) especially for the high consuming activities, i.e., GPS positioning, data transmission and wireless channel listening. Many energy preserving solutions for opportunistic network nodes have been investigated for a long time together with innovative methodologies aimed to find the optimum duty cycle for battery-equipped devices [15,30]. More specifically, techniques such as erasure codes and probabilistic network coding have proven to maximize the probability of collecting data in networks where predictable and stable paths may never exist [31]. In this way the the number of contacts needed to collect the data is reduced [32].

There are also many cases in which biological requirements calls for energy harvesting techniques. In these cases several solution can be adopted according to the type of harvesting technique selected. An extensive overview of is presented in [33] that compares sensor systems—architecture, energy sources and storage technologies. A recent overview on energy harvesting solutions is presented in [34]. They discuss how the various harvesting solutions are associated to the different operating scenarios. Authors also compare research and commercial solutions that also include an harvester component similar to the one we included in our design.

### 6.3. Comparison with Commercial Devices

Several commercial devices to track movements of animals in the wild have been made available in recent years. A systematic review of the design trade off (including the cost) is reported in [1]. Table 2 lists the main properties of some devices commercially available, for a comparison with the system presented in this paper.

The main characteristic that distinguishes our device from other products is the absence of the battery. Compliance with such a requirement has been obtained through the solution of several hardware and software challenges as discussed in the previous sections. The communication technology is rather variable, the available solutions include: cellular networks for the data collection in urban and suburban regions, satellite networks [35] such as Iridiurm [36] or Argos [37] for world wide coverage. VHF or UHF radios are chosen for local coverage associated with portable o fixed gateways deployed in the monitoring area.

Finally it is worth to note that all the commercial devices are based on a communication technology that requires a large antenna (between 17 and 20 cm) in addition to the GPS antenna. In our device, a small antenna can be used because communications occur at higher frequency. The choice of using a small antenna was in our case determined by the fact long antenna could imply a higher risk of breakage. This would be detrimental for the purposes of long-term studies of iguanas.

## 7. Conclusions

In this paper the design and the assessment of a Wireless Sensor Node specifically designed for animal monitoring has been presented. Such a device will be used to track movements of a recently discovered rare species of iguana, known as Pink Iguana (*Conolophus marthae*) endemic to the Wolf Volcano on Isabela island (Galapagos, Ecuador).

The device is build to operate in a harsh environment, without any telecommunication infrastructure available and/or power supply. To this aim, a energy harvesting solution based on a small solar power and a supercapacitor have been designed. The device is equipped with a low-power transceiver, a set of sensors (temperature, humidity, light sensor, GPS) and has been packed inside a transparent PVC enclosure to ensure protection.

The performance of the device has been measured in tests. The calibration tests of the light sensor showed that is possible to estimate the intensity of UVB radiation (an important parameter for iguanas) from the intensity of UVA radiation which is more intense and not reduced by PVC protection.

Field testing of newly developed devices in real experimental conditions are recommendable. Indeed, in future works, the technical theoretical features of the device will be evaluated also considering its performance in data collection and transmission under the real operational conditions.

## Figures and Tables

**Table 1:** Average power consumption.

State	Power Consumption	Duration	Description
*Idle*	0.054 mW	-	In the *Idle* state all the power sections are switched off. The power absorbed can be accounted to the LTC3106 and to the LDO. It is worth to note that the CC1310 is in the stand-by state, retains information on RAM consuming about 100 nA.
*GPS On*	89.1 mW	60 s	The greatest part of the power consumption is accountable to the GPS modules that absorbs about 25 mA.
*Sensor On*	0.61 mW	5 s	The main absorption is accountable to the UV sensor (0.1 mA).
*Flash On*	128.7 mW	0.1 s	The flash memory writing power absorption is about 35 mA.
*TX On*	1254 mW	0.5 s	The power consumption of transmission mode has been measured using the continuous wave.
*RX On*	29 mW	3 s	The receiving state mode absorbs about 6 mA.

**Table 2:** GPS Tracker Comparison.

Device	Company	Solar	Battery	Weight	Size mm W × L × H	Antenna Size cm	Network	Life-Time
*Solar Argos*/*GPS 17g PTT*	Microwave Telemetry [38]	yes	yes	17 g	63 × 17 × 17	18	Argos	3 years
PinPoint VHF Solar	Lotek [39]	yes	yes	6–16 g	40 × 18 × 11	17.78	VHF	6 months
*22G Solar* *GPS PTT*	Geotrack [40]	yes	yes	22 g	62 × 21 × 16	20	VHF	3 years
*GPS/Iridium* *Avian System*	Telonics [41]	no	yes	140 g	82 × 35 × 34	17	Iridium VHF	1.5 year (1 fix/day)
*This paper*	-	yes	no	17 g	76 × 22 × 16	embedded	UHF	1.5 year
